# Effects of Alirocumab and Evolocumab on Cardiovascular Mortality and LDL-C: Stratified According to the Baseline LDL-C Levels

**DOI:** 10.31083/RCM26980

**Published:** 2025-04-25

**Authors:** Hui Ma, Wenfang Ma, Yang Liu, Lixing Chen, Peng Ding

**Affiliations:** ^1^Department of Cardiology, The First Affiliated Hospital of Kunming Medical University, 650032 Kunming, Yunnan, China

**Keywords:** alirocumab, evolocumab, low-density lipoprotein cholesterol (LDL-C), baseline stratification, cardiovascular mortality, lipid-lowering efficacy

## Abstract

**Background:**

A meta-analysis was conducted to determine whether the cardiovascular mortality and lipid-lowering effects of alirocumab and evolocumab are influenced by various baseline low-density lipoprotein cholesterol (LDL-C) levels.

**Methods:**

We searched for literature published before June 2023. Eligible randomized controlled trials (RCTs) included adults treated with alirocumab or evolocumab and reported LDL-C changes and cardiovascular deaths. The primary endpoints were cardiovascular mortality and percent changes in LDL-C from baseline.

**Results:**

Forty-one RCTs were included in the meta-analysis. Evolocumab did not significantly affect the outcome of cardiovascular mortality whether the baseline data were greater than 100 mg/dL or less than 100 mg/dL. However, the stratified result showed that alirocumab decreased the risk of cardiovascular mortality in patients with a baseline LDL-C level of ≥100 mg/dL (relative risk (RR) 0.45; 95% CI: 0.22 to 0.92; *p* = 0.03). In terms of lipid-lowering efficacy, alirocumab (mean difference (MD) –56.62%; 95% CI: –60.70% to –52.54%; *p* < 0.001) and evolocumab (MD –68.10%; 95% CI: –74.85% to –61.36%; *p* < 0.001) yielded the highest percentage reduction in LDL-C level when baseline levels were 70–100 mg/dL, while the smallest reduction in alirocumab (MD –37.26%; 95% CI: –44.06% to –30.46%; *p* < 0.001) and evolocumab (MD –37.55%; 95% CI: –40.47% to –34.63%; *p* < 0.001) occurred with baseline LDL-C levels of ≥160 mg/dL.

**Conclusions:**

Alirocumab and evolocumab presented a better lipid-lowering effect when the baseline LDL-C levels were <100 mg/dL. Alirocumab was associated with a significant reduction in cardiovascular mortality at baseline LDL-C levels of ≥100 mg/dL. This finding can have significant implications for the development of personalized drug therapy.

**The PROSPERO Registration:**

CRD42023446723, https://www.crd.york.ac.uk/PROSPERO/view/CRD42023446723.

## 1. Introduction

Aggressive lipid management in high-risk cardiovascular (CV) patients can significantly improve cardiovascular outcomes. Statins represent the foundation of clinical lipid management. Nevertheless, for patients who are incapable to attain the targeted low-density lipoprotein cholesterol (LDL-C) levels with intensive statin therapy or for patients who are intolerant to statins, a combination of PCSK9 mAbs (proprotein convertase subtilisin/kexin type 9 monoclonal antibodies) may be employed as an alternative [1, 2]. The PCSK9 is a protein that reduces the ability of liver cells to clear LDL-C from the blood by binding to the LDL-C receptor on the surface of liver cells and promoting its degradation, thereby increasing the level of LDL-C in the blood [3, 4]. PCSK9 mAbs can inhibit the degradation of LDL-C receptors by PCSK9, thereby increasing the number of LDL-C receptors present on the surface of hepatocytes and facilitating their binding to LDL-C, which in turn reduces the level of LDL-C in the blood [5]. The current list of approved PCSK9 monoclonal antibodies includes alirocumab and evolocumab. Both alirocumab and evolocumab are fully human monoclonal antibodies, and the technical platforms are VelocImmune and XenoMouse, respectively. Alirocumab and evolocumab are frequently employed in patients who have exhibited suboptimal responses to conventional lipid-lowering regimens, such as those with hypercholesterolemia or familial hypercholesterolemia (FH). FH is a monogenic autosomal inherited disorder of cholesterol metabolism. FH genotypes can be divided into four types: heterozygous FH (HeFH), homozygous FH (HoFH), compound HeFH and double HeFH. Among them, HeFH is the most common, with an estimated prevalence of 1/250~1/200. Before treatment, HeFH patients contain high levels of free PCSK9 in their plasma [6]. High levels of free PCSK9 cause the degradation of LDL receptor (LDLR) on the surface of hepatocytes, leading to a decrease in LDLR. The function of PCSK9 inhibitors is to increase the LDLR on the liver surface by reducing PCSK9 levels, thereby increasing the clearance rate of LDL-C and achieving a significant lipid-lowering effect. The direct reason for the lack of receptors in HeFH patients is the high level of free PCSK9 in plasma. With the use of inhibitors, the amount of LDLR increases, thus increasing the biological effect of the liver in clearing LDL-C from the circulation. Most HeFH patients are intolerant to statins but have a ≥50% reduction in LDL-C after treatment with PCSK9 inhibitors. In subjects with FH, PCSK9 mAbs have a greater lipid-lowering effect in HeFH than in HoFH [7].

A substantial body of evidence has demonstrated that statins are an effective intervention for reducing the incidence of cardiovascular events. Moreover, the combination of ezetimibe or PCSK9 mAbs with intensive statin therapy has been evidenced to result in a further reduction of LDL-C levels, thereby further declining cardiovascular risk. Among these, alirocumab was more significant in the reduction of cardiovascular death and was related to baseline LDL-C [1, 8]. The ODYSSEY OUTCOMES demonstrated that the efficacy of alirocumab in reducing the incidence of endpoint events was more pronounced in subjects with baseline LDL-C levels of 100 mg/dL or above [1]. However, the FOURIER trial did not observe an impact of evolocumab on cardiovascular mortality in individual outcomes [2]. It is unclear whether the baseline level of LDL-C affects this result. A comprehensive meta-analysis showed that mortality reduction was only observed in trials with patients who had mean baseline LDL-C levels higher than 100 mg/dL, and all-cause mortality was not related to the achieved targeted LDL-C levels [9]. Another meta-analysis reported that a reduction in cardiovascular mortality occurred in trials with patients who had baseline LDL-C levels greater than 130 mg/dL, and trials reducing LDL-C by more than 50% did not consistently result in further decreases in all-cause and cardiovascular mortality [10]. The current research mainly elaborated on the association between less/more intensive LDL-C–lowering therapy and cardiovascular mortality, and the benefits of alirocumab and evolocumab on cardiovascular mortality in patients with various baseline LDL-C levels are unclear. To better evaluate the association between PCSK9 mAbs and cardiovascular mortality, we conducted a subgroup analysis according to baseline LDL-C levels and drug types and investigated the effects of the different drugs on cardiovascular mortality as well as their lipid-lowering efficacy in patients with various baseline LDL-C levels.

## 2. Methods

### 2.1 Data Sources and Search Strategy

The methods of this meta-analysis were based on the Cochrane Handbooks [11] and the PRISMA (Preferred Reporting Items for Systematic reviews and Meta-Analyses) statement [12]. We registered it with PROSPERO (CRD42023446723).

Two independent investigators (HM and YL) conducted a comprehensive search of PubMed, Ovid, Embase, and ClinicalTrials.gov for articles published prior to June 2023. The key words of retrieval were “Proprotein convertase subtilisin/kexin type 9 inhibitor” OR “PCSK9 inhibitor” OR “PCSK9 monoclonal antibodies” OR “PCSK9 mAbs” OR “Alirocumab” OR “Evolocumab” OR “REGN727” OR “SAR236553” OR “AMG145” OR “RN316” OR “PF04950615” OR “IBI306”. In addition, we avoided possible omissions of eligible studies by searching the references of the review articles. Any points of contention were resolved through deliberation until a unanimous decision was attained. The decision regarding the ultimate resolution of the discrepancy was made by the corresponding author.

### 2.2 Eligibility Criteria

The trials were eligible for inclusion when they satisfied the following criteria: (1) population: adult patients with hypercholesterolemia or HeFH at high cardiovascular risk; (2) intervention: patients were treated with alirocumab or evolocumab; (3) control: patients who received other standard lipid-lowering drugs or placebo; (4) outcomes: percent changes in LDL-C from baseline, incidence of cardiovascular deaths; and (5) study design: phase II or III RCTs (randomized controlled trials). The quality of each included trial was assessed in accordance with the criteria set out in the Cochrane Collaboration guidelines [11].

### 2.3 Study Endpoints

The primary endpoints were cardiovascular mortality and percent changes in LDL-C from baseline. Cardiovascular death included death resulting from an acute myocardial infarction (MI), sudden cardiac death, death due to heart failure (HF), death due to stroke, death due to cardiovascular procedures, death due to cardiovascular haemorrhage, and death due to other cardiovascular causes. See Table 1 for the specific definition of cardiovascular death in each trial. Regarding percentage changes in LDL-C levels from baseline, directly measured LDL-C values were prefered extract when both measured and calculated LDL-C levels were reported in a trial [13].

**Table 1.** **The definition of cardiovascular death in various trials**.

Trial (Clinical Trials ID)	Definition of cardiovascular death
ODYSSEY J-IVUS	One patient in the standard-of-care arm died during the TEAE period from sepsis, acute coronary syndrome, and cardiac failure.
(NCT02984982)	
ODYSSEY OUTCOMES	Death from cardiovascular causes (coronary heart disease, cardiac failure, arrhythmia, myocardial/pericardial disease, etc).
(NCT01663402)	
ODYSSEY COMBO I	Coronary heart disease (including undetermined cause).
(NCT01644175)	
ODYSSEY COMBO II	Coronary heart disease (including undetermined cause).
(NCT01644188)	
ODYSSEY DM-INSULIN	TEAEs leading to death (no death case).
(NCT02585778)	
ODYSSEY EAST	Coronary heart disease (including undetermined cause)^a^.
(NCT02715726)	
ODYSSEY FH I	Three cardiovascular deaths occurred in the alirocumab group, one due to acute myocardial infarction; two classified as due to sudden cardiac death (congestive cardiac failure and coronary artery disease for the first death, and myocardial infarction for the second).
(NCT01623115)	
ODYSSEY LONG TERM	Death from coronary heart disease, including death from unknown cause.
(NCT01507831)	
ODYSSEY OPTIONS I	Cardiac arrest occurred in one patient (control group) with a history of acute myocardial infarction.
(NCT01730040)	
ODYSSEY OPTIONS II	One patient who was randomized to the control group died of a subdural hematoma during the course of the study; the death was adjudicated as a cardiovascular death.
(NCT01730053)	
FOURIER	Cardiovascular death: due to acute myocardial infarction, stroke and other cardiovascular death.
(NCT01764633)	
GLAGOV (NCT01813422)	Death from cardiovascular events.
BANTING (NCT02739984)	Sudden cardiac death 8 days after exposure to evolocumab in one patient, not considered related to evolocumab by investigator.
DESCARTES (NCT01516879)	The two deaths were from cardiac failure and myocardial infarction.
EVOPACS (NCT03287609)	A patient with a history of non-ST-segment elevation myocardial infarction died of cardiogenic shock. Another patient with a history of atrial fibrillation, who had many heart operations, died of progressive cardiogenic shock and multi-organ failure.
LAPLACE-2 (NCT01763866)	One death was reported during the study in a patient receiving rosuvastatin and subcutaneous placebo.
OSLER (1&2) (NCT01439880/NCT01854918)	Cardiovascular death includes death resulting from an acute myocardial infarction, sudden cardiac death, heart failure, and stroke, death due to cardiovascular procedures, death due to cardiovascular hemorrhage, and death due to other cardiovascular causes.

TEAE, treatment-emergent adverse event. ^a^, following adjudication review, primary causes of death were reported of cardiovascular origin in 2 patients in the alirocumab group vs 2 patients in the control group, including 1 vs 2 patients with a primary cause as coronary heart disease death. Related clinical trial information query: https://clinicaltrials.gov/.

### 2.4 Data Extraction

Data were independently extracted by two authors (HM and YL), and any divergences were settled via the corresponding author. The information we extracted from the various studies was as follows: title of trials, date of publication, the registration number of clinical trial, baseline LDL-C mean, doses of alirocumab and evolocumab, the information of control group, background lipid-lowering treatment, length of follow-up for blood lipids and adverse events, mean age, the proportion of patients with diabetes mellitus, the ratio of patients with coronary heart disease, and patient characteristics.

For cardiovascular death events, we extracted the total amount of participants and the number of cardiovascular deaths from the studies. For the percent changes in LDL-C from baseline, we extracted the mean, standard deviation (SD), and the number of participants in each group. In the absence of reported SDs, these were calculated from the standard error or 95% confidence interval (CI).

### 2.5 Statistical Analysis

Analyses were conducted using Review Manager 5.4 (Cochrane Collaboration, Copenhagen, Denmark). Current guidelines recommend that patients should be classified into various treatments groups according to their blood lipid levels. Different regions have different grouping strategies. The benefits of lipid-lowering therapy vary in patients with different risk stratification. In the ODYSSEY OUTCOMES trial, we found that the absolute reduction in the risk of the composite primary endpoint with alirocumab was greatest in patients with baseline LDL-C ≥100 mg/dL [1]. This baseline level also corresponds to the high-risk group in the stratification strategy. However, this conclusion was based on a stratification strategy for multiple outcomes, and the observed reduction in all-cause mortality was labeled “nominal significant”, which makes interpretation unclear [14]. Therefore, we took the cardiovascular mortality outcome event out alone for stratified analysis. For research on cardiovascular mortality, we performed subgroup analysis according to drug type (alirocumab and evolocumab) and baseline LDL-C level (baseline LDL-C <100 mg/dL and ≥100 mg/dL). For the percent changes in LDL-C from baseline, studies were grouped into four subgroups according to baseline LDL-C level (baseline LDL-C <100 mg/dL, ≥100 and <130 mg/dL, ≥130 and <160 mg/dL, ≥160 mg/dL).

Relative risks (RRs) and 95% CIs were applied for categorical variables (cardiovascular mortality). The mean difference (MD) and 95% CI of the percent change from baseline were utilised. The pooled effect estimates ascertain whether a fixed-effects or a random-effects model would be more appropriate by the test of heterogeneity. And then, the most suitable statistical method (the employment of common statistical methods in two models are detailed in **Supplementary Material-V1** was then selected in accordance with the selected model. Among them, inverse variance method can be used to combine binary data and continuous data, and can handle various effect values. However, Mantel-Hanenszel (M-H) method is more robust when there are fewer studies and the incidence of study events is low, but M-H method can only handle binary data. Peto method is an improvement of M-H method, which can only be used to deal with OR (odds ratio) values, especially when the incidence of events in the study is very low. However, this rule should not be used if the treatment effect is very large or if the sample size of the experimental and control groups in the study is severely unbalanced. The DerSimonian-Laird (D-L) method is a statistical method for random effects model, which is applicable to various effect values. However, it often gives greater weight to small sample studies, which often have publication bias. Therefore, this method may sacrifice the evidence of high-quality studies to emphasize small sample studies. The selection of model is made according to the significance of the heterogeneity test (Q-test). The random effects model will be used when the Q-test is significant (I-squared ≥50%, or *p* < 0.05), and the fixed effects model will be used when it is not significant (I-squared <50% and *p* ≥ 0.05). The inspection level for pooled results was two-sided, and *p* < 0.05 was considered to indicate statistical significance. Heterogeneity was evaluated using the chi-square heterogeneity statistic with *p* < 0.05 considered to indicate statistical significance, and I-squared >50% was considered to exist heterogeneity [15]. The quality of RCTs was evaluated by the Cochrane Cooperative Network Bias Risk Assessment Tool, which includes seven criteria: random sequence generation (selection bias), allocation concealment (selection bias), blinding of participants and personnel (performance bias), blinding of outcome assessment (detection bias), incomplete outcome data (attrition bias), selective reporting (reporting bias), and other bias [11].

### 2.6 Data Accessibility and Ethical Statement

All the data we extracted can be obtained from ClinicalTrials.gov and other published literature. All trials included in this paper stated that the protocol had been approved by ethics committee the or relevant institutional review board. All participants provided the written informed consent prior to their involvement in the study.

## 3. Results

### 3.1 Literature Screening

**Fig. 1.**
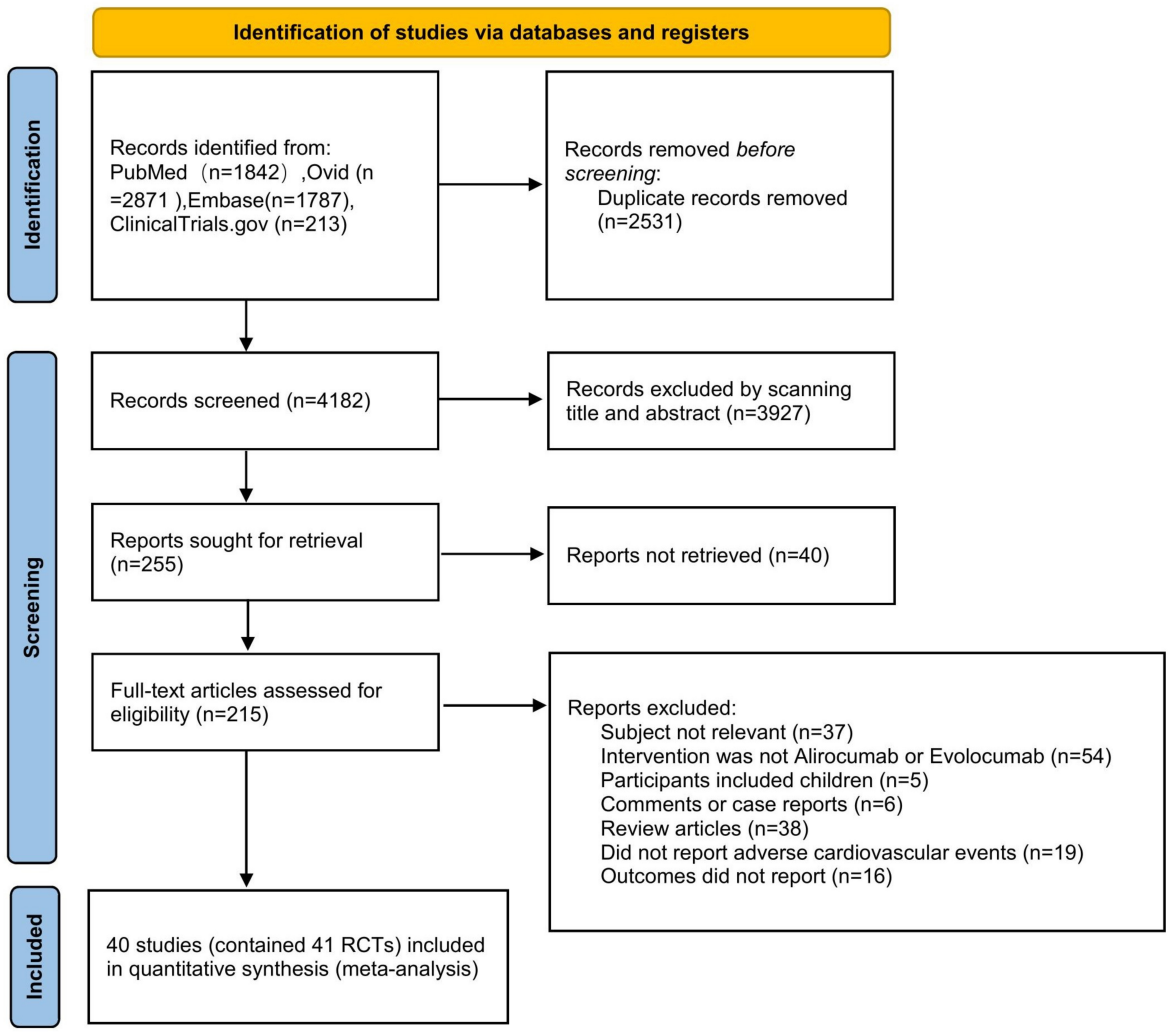
**The Preferred Reporting Items for Systematic reviews and Meta-Analyses (PRISMA) flow diagram**. ‘Subject not relevant’ means that Alirocumab or Evolocumab appears in the abstract or text of the article, but the research focus of the article is not on these two drugs, which may only appear in the article as a treatment or intervention. RCT, randomized controlled trials.

A total of 6713 records were retrieved from PubMed, Ovid, Embase, and ClinicalTrials.gov. A total of 215 records were accessed via full-text perusal after discarding duplicate records and removing irrelevant articles by scanning titles and abstracts. A total of 175 publications were excluded for the following reasons: the subject was not relevant (n = 37); the intervention treatment did not include alirocumab or evolocumab (n = 54); the participants included children (n = 5); the publications were comments or case reports (n = 6) or review articles (n = 38); and adverse cardiovascular events (n = 19) or outcomes (n = 16) were not reported. Finally, the meta-analysis was based on 40 studies, comprising 41 RCTs (Fig. 1).

### 3.2 Characteristics of Included Trials and Patients

Table 2 lists 41 RCTs included in the study. These RCTs were published between 2012 and 2020. The mean range of baseline LDL-C levels was 2.4 to 5.69 mmol/L (92.8 to 219.9 mg/dL), and further subgroup analysis was performed according to baseline levels. In 23 RCTs from 22 articles [1, 8, 16, 17, 18, 19, 20, 21, 22, 23, 24, 25, 26, 27, 28, 29, 30, 31, 32, 33, 34, 35], patients received alirocumab; among them, 10 RCTs [1, 8, 19, 21, 23, 24, 27, 31, 33, 35] reported cardiovascular deaths. Evolocumab was given in 18 RCTs [2, 36, 37, 38, 39, 40, 41, 42, 43, 44, 45, 46, 47, 48, 49, 50, 51, 52] of these, 7 trials [2, 39, 41, 45, 47, 48, 49] provided data for cardiovascular events. Regarding the lipid-lowering effect of alirocumab and evolocumab, RCTs were divided into four layers according to the baseline LDL-C level. It was emphasized that the baseline LDL-C levels were different at various dosages in the same trial; hence, the same RCT appeared in different baseline stratifications in the following analysis. The follow-up period of blood lipids ranged from 8 to 192 weeks, while that for the evaluation of cardiovascular events spanned from 8 to 144 weeks across the included trials. The mean weighted age for participants across primary studies ranged from 49.6 to 64.4 years, and the proportions of patients with coronary heart disease (CHD) and diabetes mellitus (DM) were 3–100% and 0.16–100%, respectively. Most participants were diagnosed with hypercholesterolemia or heterozygous familial hypercholesterolemia, and the included patients of 2 RCTs were diagnosed with acute coronary syndrome [35, 48]. Background therapy was added with stable statin or other lipid-lowering therapy in most of the RCTs. The OSLER [45] study integrated data from OSLER-1 and OSLER-2. In addition, ODYSSEY FH I and ODYSSEY FH II were reported in one article [24].

**Table 2.** **Baseline characteristics of randomized controlled trials**.

Trial	Year	Clinical Trials ID	Baseline LDL-C Mean mmol/L (mg/dL)^a^	PCSK9 mAbs	Controls	Background therapy^c^	Follow-up (Lipid/AEs) (weeks)	Age (year)	CHD (%)	DM (%)	Patients
STEIN	2012	NCT01266876	4.0 (154.7)	ALI 150 mg, 200 mg or 300 mg Q4w; or 150 mg Q2w	Placebo	Stable statin with/without ezetimibe	12/12 w	53.4	42	4.0	HeFH with/without CV events
Roth *et al*.	2012	NCT01288469	3.28 (126.9)	ALI 150 mg Q2w	Placebo	Stable atorvastatin 10/80 mg	8/8 w	56.9	3	14	HC with/without CV risk elements
Mckenney *et al*.	2012	NCT01288443	3.19 (123.2)	ALI 200 mg or 300 mg Q4w;	Placebo	Stable dose of atorvastatin	12/12 w	56.7	10	22	HC with CV high-risk elements
				Or 50, 100, 150 mg Q2w							
RUTHERFORD	2012	NCT01375751	4.1 (158.5)	EVO 350 mg or 420 mg Q4w	Placebo	Stable dose of statin	12/12 w	49.6	21	NA	HeFH with/without CV events
MENDEL	2012	NCT01375777	3.2 (123.7)	EVO 70 mg or 105 mg or 140 mg Q2w; 280 mg or 350 mg or 420 mg Q4w	Eze/Placebo	None	12/12 w	50.6	8	0.2	HC with CV high-risk elements
LAPLACE-TIMI 57	2012	NCT01380730	3.2 (123.7)	EVO 70 mg or 105 mg or 140 mg Q2w; 280 mg or 350 mg or 420 mg Q4w	Placebo	Stable dose of statin	12/12 w	60.2	30	16	HC with CV high-risk elements
YUKAWA	2014	NCT01652703	3.7 (143.1)	EVO 70/140 mg Q2w; or 280/420 mg Q4w	Placebo	Stable statin with/without other lipid-modifying therapy	12/12 w	61.5	25.1	38.1	HC with CV high-risk elements
DESCARTES	2014	NCT01516879	2.69 (104.0)	EVO 420 mg Q4w	Placebo	Lipid-lowering therapy	52/52 w	56.2	15.1	11.5	HC with CHD risk factors
GAUSS-2	2014	NCT01763905	4.97 (192.0)	EVO 140 mg Q2w or 420 mg Q4w	Eze	Not on statin/Other	12/12 w	61.5	32.2	20.2	Statin-intolerant
MENDEL-2	2014	NCT01763827	3.67/3.72	EVO 140 mg Q2w or 420 mg Q4w	Placebo/Eze	None	12/12 w	53.3	9.9	0.16	HC with CV risk factors
			(142.0/144.0)^b^								
LAPLACE-2	2014	NCT01763866	2.84 (109.7)	EVO 140 mg Q2w or 420 mg Q4w	Placebo/Eze	Moderate or high intensity statin therapy	12/12 w	59.6	22.5	15.5	Primary HC and mixed dyslipidemia
ODYSSEY COMBOII	2015	NCT01644188	2.8 (108.3)	ALI 75 mg Q2w	Eze 10 mg QD	Stable statin lack of other lipid-lowing treatments	24/58 w	61.6	90.1	31.0	HC with CV high-risk elements
ODYSSEY ALTERNATIVE	2015	NCT01709513	4.7 (181.7)	ALI 75 mg Q2w	Eze 10 mg QD	None	24/34 w	63.4	46.5	23.9	Statin-intolerant HC with CV high-risk elements
ODYSSEY OPTIONSI	2015	NCT01730040	2.7 (104.4)	ALI 75 mg or 150 mg Q2w	Eze10 mg QD; double ATV dose; change to RSV 40 mg QD.	Stable atorvastatin 20/40 mg QD	24/32 w	62.9	56.3	49.9	HC with high-risk CV factors
ODYSSEY LONG TERM	2015	NCT01507831	3.2 (123.7)	ALI 150 mg Q2w	Placebo	High/maximum tolerated dose Statin with/without other lipid-lowing interventions	24/86 w	60.5	68.6	34.6	HC with high-risk CV factors
ODYSSEY COMBOI	2015	NCT01644175	2.6 (100.5)	ALI 75 mg Q2w	Placebo	Stable-statin with/without other lipid-lowing treatments	24/60 w	63	78.2	43.0	HC with high-risk CV factors
ODYSSEY FHI	2015	NCT01623115	3.6 (139.2)	ALI 75 mg Q2w	Placebo	Stable-statin with/without other lipid-modifying treatments	24/34 w	51.9	46.3	11.7	HeFH with/without CV events
ODYSSEY FHII	2015	NCT01709500	3.6 (139.2)	ALI 75 mg Q2w	Placebo	Stable-statin with/without other lipid-lowing treatments	24/34 w	53.2	35.7	4.0	HeFH with/without CV events
ODYSSEY MONO	2015	NCT01644474	3.62 (139.7)	ALI 75/150 mg Q2w	Eze	None	24/34 w	60.2	NA	3.9	HC
RUTHERFORD-2	2015	NCT01763918	4.02 (155.5)	EVO 420 mg Q4w or 140 mg Q2w	Placebo	Stable-statin with/without other lipid-modifying treatments	12/14 w	51.1	31.3	NA	HeFH with/without CV events
OSLER (1&2)	2015	NCT01439880	3.12 (120.5)	EVO 140 mg Q2w/420 mg Q4w+standard treatments	Standard treatments	Standard-therapy based on local guidelines	48/48 w	58.0	20.1	13.4	HeFH/HC with/without CV events
		NCT01854918									
ODYSSEY OPTIONSII	2016	NCT01730053	2.8 (108.3)	ALI 75 mg Q2w	Ezetimibe 10 mg QD; or double RSV dose	Rosuvastatin 10/20 mg QD	24/34 w	60.95	58	41.3	HC with high-risk CV elements
ODYSSEY HIGH FH	2016	NCT01617655	5.12 (197.8)	ALI 150 mg Q2w	Placebo	Stable-statin with/without other lipid-lowing therapy	24/34 w	50.6	49.5	14.0	HeFH with/without CV events
ODYSSEY JAPAN	2016	NCT02107898	3.7 (143.1)	ALI 75 mg Q2w	Placebo	Stable-statin with/without other lipid-lowing treatments	24/52 w	60.8	21.3	68.5	HC with high-risk CV elements
ODYSSEY ESCAPE	2016	NCT02326220	4.7 (181.7)	ALI 150 mg Q2w	Placebo	Lipid-lowing treatments with/without stable-statin	18/28 w	58.7	79	NA	HeFH with/without CV events
ODYSSEY CHOICEI	2016	NCT01926782	2.91 (112.4)	ALI 75 mg Q2w/300 mg Q4w	Placebo	With or without statin/Other	24/56 w	60.8	NA	27.0	HC with moderate-risk to very-high-risk CV elements
ODYSSEY CHOICEII	2016	NCT02023879	4.2 (162.4)	ALI 75 mg Q2w/150 mg Q4w	Placebo	Not on statin/Other	24/32 w	63.1	49.8	16.3	HC with moderate-risk to very-high-risk CV elements
YUKAWAII	2016	NCT01953328	2.7 (104.4)	EVO 420 mg Q4w or 140 mg Q2w	Placebo	ATV 5/20 mg QD	12/12 w	61.5	12.9	48.8	HC with high-risk CV factors
GAUSS-3	2016	NCT01984424	5.69 (219.9)	EVO 420 mg Q4w	Eze 10 mg QD	None	24/24 w	58.8	31.7	11.9	Statin-intolerant with CHD/ CV risk factors
GLAGOV	2016	NCT01813422	2.4 (92.8)	EVO 420 mg Q4w	Placebo	Stable-statin/Eze with/without other lipid-lowing treatments	76/78 w	59.8	100	20.9	Occurrence of CV events with high-risk CV elements
ODYSSEY DM-INSULIN	2017	NCT02585778	2.9 (112.1)	ALI 75 mg Q2w	Placebo	Lipid-lowing treatments with/without stable-statin	24/32 w	62.8	31.9	100	Insulin-treatment T1/T2DM with high-risk CV elements
FOURIER	2017	NCT01764633	2.4 (92.8)	EVO 420 mg Q4w or 140 mg Q2w	Placebo	ATV 20 mg QD or equivalent with/without Eze	48/144 w	62.5	81.1	36.6	Occurrence of CV events in HC
ODYSSEY-KT	2018	NCT02289963	2.5 (96.7)	ALI 75 mg Q2w	Placebo	Stable-statin with/without other lipid-lowing therapy	24/32 w	60.1	95.98	35.2	HC with high-risk CV elements
ODYSSEY OUTCOMES	2018	NCT01663402	2.4 (92.8)	ALI 75 mg rise to 150 mg Q2w	Placebo	Lipid-lowing treatments with/without stable-statin	192/134 w	58.6	100	28.8	Occurrence of CV events in HC
ODYSSEY EAST	2019	NCT02715726	2.9 (112.1)	ALI 75 mg rise to 150 mg Q2w	Eze 10 mg QD	Stable statin	24/34 w	58.6	97.6	27.5	HC with high-risk CV elements
ODYSSEY CHOICE I-DM SUBGROUP	2019	NCT01926782	2.8 (108.3)	ALI 300 mg Q4w or 75 mg Q2w	Placebo	Stable-statin or other lipid-lowing intervention	24/58 w	62.7	59.6	100	T2DM with high-risk CV elements
ODYSSEY J-IVUS	2019	NCT02984982	2.51 (96.9)	ALI 75 mg rise to 150 mg Q2w	Standard of care	Stable-dose statin therapy	36/39 w	60.9	12.1	31.9	Acute coronary syndrome
BANTING	2019	NCT02739984	2.82 (109.2)	EVO 420 mg Q4w	Placebo	Stable-statin or other lipid-modifying therapy	12/12 w	62.5	38.7	100	T2DM with high-risk CV elements
BERSON	2019	NCT02662569	2.4 (92.8)	EVO 420 mg Q4w or 140 mg Q2w	Placebo	ATV 20 mg QD	12/14 w	62	29.4	100	T2DM with high-risk CV elements
EVOPACS	2019	NCT03287609	3.52 (136.1)	EVO 420 mg Q4w	Placebo	ATV 40/80 mg QD	8/8 w	60.8	100	15.3	Acute coronary syndrome
GAUSS-4	2020	NCT02634580	4.89 (189.0)	EVO 420 mg Q4w or 140 mg Q2w	Eze 10 mg QD	None	12/12 w	64.4	39.3	18.0	Statin-intolerant HC

ALI, alirocumab; ATV, atorvastatin; CV, cardiovascular; CHD, coronary heart disease; DM, diabetes mellitus; EVO, evolocumab; Eze, ezetimibe; HC, hypercholesterolemia; HeFH, heterozygous familial hypercholesterolemia; LDL-C, low-density lipoprotein cholesterol; NA, not available; PCSK9 mAbs, proprotei-convertase-subtilisin/kexin-type 9 monoclonal antibodies; RSV, rosuvastatin; T1DM, type-1 diabetes mellitus; T2DM, type-2 diabetes mellitus; AEs, adverse events; w, weeks; Q2w, once every two weeks; Q4w, once every four weeks; ASCVD, atherosclerotic cardiovascular disease; QD, once daily. ^a^ LDL-C values converted from mg/dL to mmol/L via multiplication by 0.02586. Represents the overall baseline level, the baseline LDL-C levels were different at various dosages in the same trial. ^b^ Represents the baseline data compared with placebo and ezetimibe respectively. ^c^ Background therapeutic regimen was maintained throughout during the study both in the experimental and control groups. ^d^ Patients at high cardiovascular risk were defined as follows: (1) patients who had previous cardiovascular events (secondary prevention); (2) patients who have not previously experienced cardiovascular events but who have high cardiovascular risk factors, including T1DM/T2DM (type 1 or type 2 diabetes mellitus), moderate chronic kidney disease (estimated glomerular filtration rate, ≥30 and <60 mL/min/1.73 m^2^ of body-surface area), severe hypercholesterolemia (LDL-C ≥190 mg/dL [≥4.9 mmol/L]), or familial hypercholesterolemia (FH) together with or without cardiovascular risk-enhancing factors (high-risk primary prevention group); or (3) patients without previous cardiovascular events but with a high 10-year cumulative risk of hard ASCVD events, which were assessed by estimation systems, such as NCEP ATP III (National Cholesterol Education Program Adult Treatment Panel III, USA), PCE (Pooled Cohort Equations, USA), JAS (Japan Atherosclerosis Society), Systematic Coronary Risk Estimation, Europe (SCORE) and others (the high-risk primary prevention group). Related clinical trial information query: https://clinicaltrials.gov/.

### 3.3 Cardiovascular Mortality

#### 3.3.1 Stratified by Drug Type (Alirocumab and Evolocumab)

**Fig. 2.**
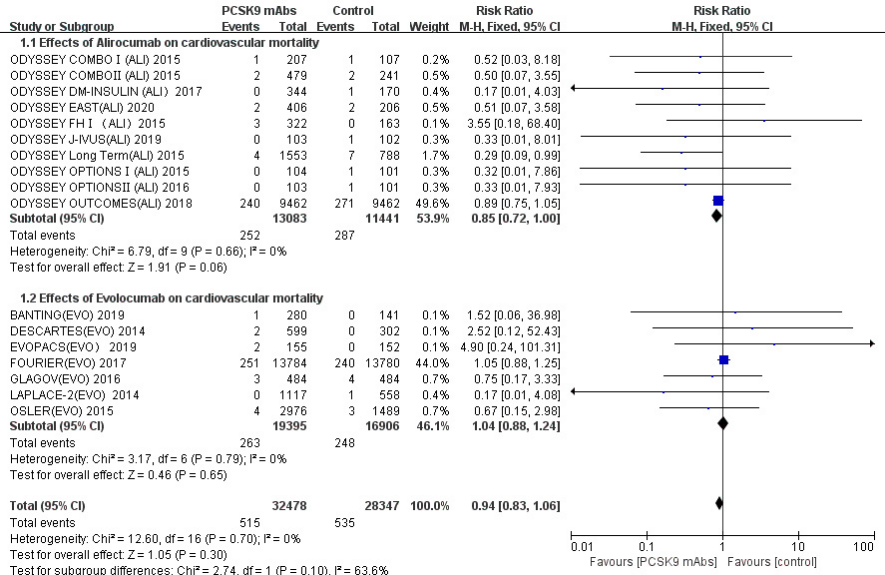
**Cardiovascular mortality stratified by drug type (alirocumab and evolocumab)**. There are currently 17 studies reporting the effects of PCSK9 inhibitors on cardiovascular mortality, including 10 studies on Alirocumab and 7 studies on Evolocumab. The overall heterogeneity test was not significant (*p* = 0.70 >0.05, I-square = 0% <50%), and the pooled effect value used the Mantel-Hanenszel (M-H) method of the fixed effect model.

Seventeen RCTs reported the incidence of cardiovascular deaths (Fig. 2). Overall, PCSK9 mAbs (alirocumab and evolocumab) were not associated with a significant change in the cardiovascular mortality (relative risk [RR] 0.94; 95% CI 0.83 to 1.06; *p* = 0.30). As shown in the two subgroups, alirocumab did not present a significant effect in the outcome of cardiovascular mortality (RR 0.85; 95% CI 0.72 to 1.00; *p* = 0.06). However, the result of quantitative synthesis showed that it was more inclined to the intervention group. Cardiovascular deaths occurred in 1.93% (252/13,083) of participants in the alirocumab group and 2.51% (287/11,441) in the control group. Alirocumab exhibited a lower incidence of cardiovascular mortality. Evolocumab had no significant effect on cardiovascular mortality (RR 1.04; 95% CI 0.88 to 1.24; *p* = 0.65). No significant heterogeneity was observed across all trials (*p* = 0.70; I-square = 0%).

In view of the potential influence of baseline LDL-C levels on the efficacy of alirocumab on cardiovascular events in ODYSSEY trials, we conducted a further analysis for baseline LDL-C <100 mg/dL and ≥100 mg/dL, respectively. The baseline data were stratified according to the ODYSSEY OUTCOMES trial.

#### 3.3.2 Stratified by Baseline LDL-C Level (<100 mg/dL and ≥100 mg/dL)

3.3.2.1 Alirocumab

**Fig. 3.**
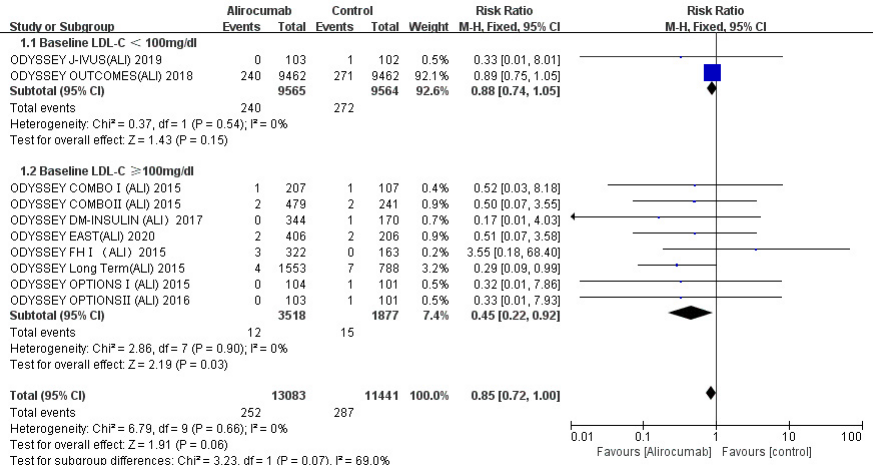
**Cardiovascular mortality of alirocumab stratified by baseline LDL-C level**. The overall heterogeneity test was not significant (*p* = 0.66 >0.05, I-square = 0% <50%), and the pooled effect value used the M-H method of the fixed effect model. LDL-C, low-density lipoprotein cholesterol.

As shown in Fig. 3, cardiovascular mortality was markedly associated with a reduction in risk only in the trials with patients who had baseline LDL-C levels of 100 mg/dL or greater (RR 0.45; 95% CI 0.22 to 0.92; *p* = 0.03), *p* value < 0.05. The result is consistent with the previous research hypothesis. Cardiovascular deaths occurred in 0.34% (12/3518) of participants in the alirocumab group and 0.80% (15/1877) in the control group when LDL-C levels were 100 mg/dL or greater. Regarding the outcome for the patients with baseline LDL-C levels less than 100 mg/dL, the intervention group did not experience superior reductions in cardiovascular-mortality compared with the control group (RR 0.88; 95% CI 0.74 to 1.05; *p* = 0.15), *p* value > 0.05. No marked heterogeneities were discovered (*p* = 0.66; I-square = 0%).

3.3.2.2 Evolocumab

**Fig. 4.**
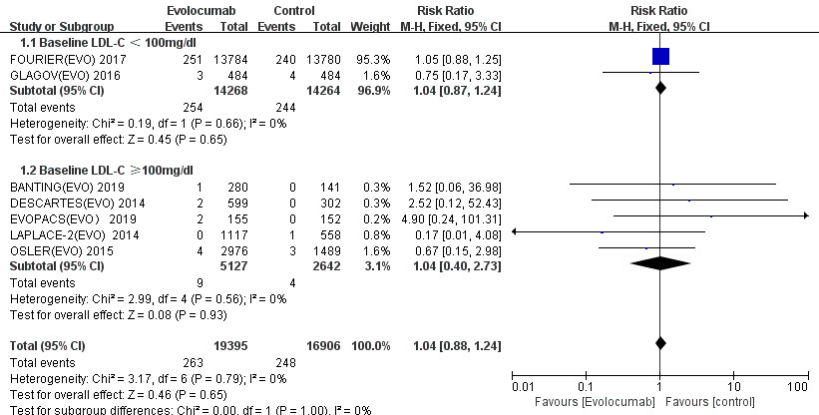
**Cardiovascular mortality of evolocumab stratified by baseline LDL-C level**. The overall heterogeneity test was not significant (*p* = 0.79 >0.05, I-square = 0% <50%), and the pooled effect value used the M-H method of the fixed effect model. LDL-C, low-density lipoprotein cholesterol.

We also stratified evolocumab based on baseline data, and there was still no difference in cardiovascular mortality whether the baseline data were greater than 100 mg/dL (RR 1.04; 95% CI 0.40 to 2.73; *p* = 0.93 >0.05) or less than 100 mg/dL (RR 1.04; 95% CI 0.87 to 1.24; *p* = 0.65 >0.05) (Fig. 4).

### 3.4 Outcome of Percent Changes in LDL-C from Baseline Stratified by Baseline LDL-C Level

**Fig. 5.**
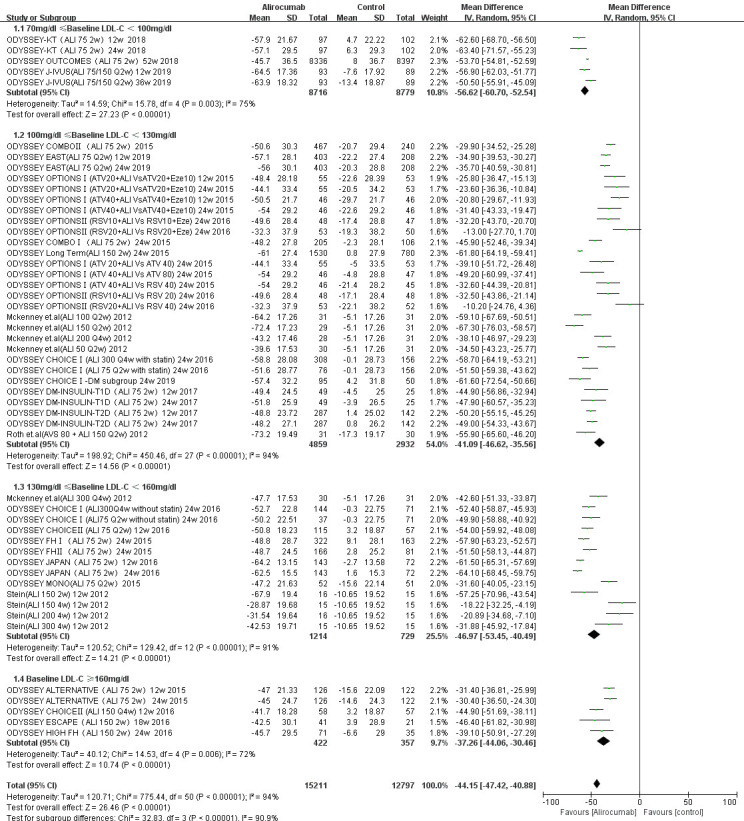
**Percent changes in LDL-C from baseline of alirocumab stratified by baseline LDL-C level**. The overall heterogeneity test was significant (*p* < 0.001, I-square = 94% >50%), and the pooled effect value used the inverse variance (IV) method of the random effects model.

**Fig. 6.**
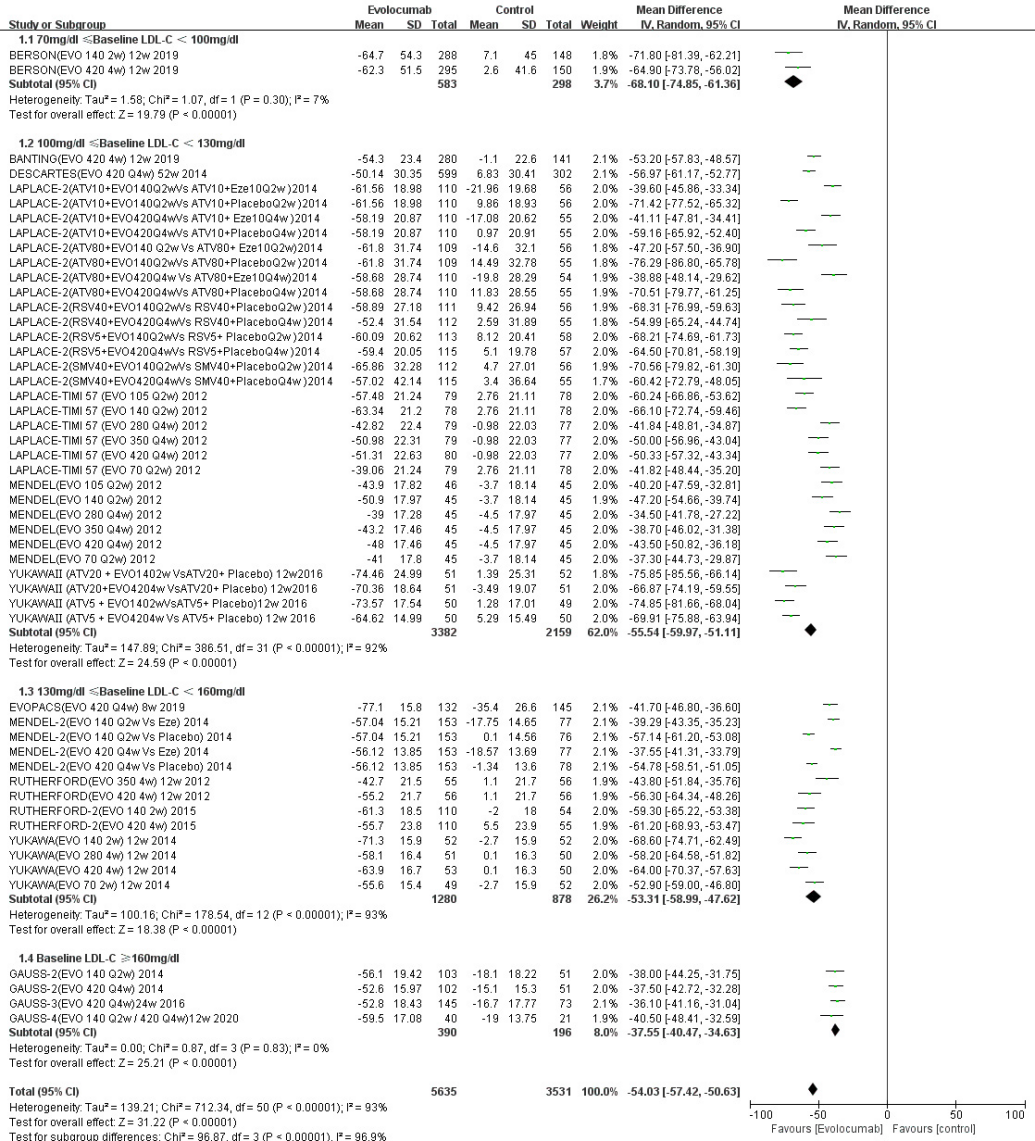
**Percent changes in LDL-C from baseline of evolocumab stratified by baseline LDL-C level**. The overall heterogeneity test was significant (*p* < 0.001, I-square = 93% >50%), and the pooled effect value used the Inverse variance method of the random effects model. SMV, simvastatin.

Figs. 5,6 show that alirocumab (MD –44.15%; 95% CI –47.42% to –40.88%; *p* < 0.001) and evolocumab (MD –54.03%; 95% CI –57.42% to –50.63%; *p* < 0.001) had significant efficacy in reducing LDL-C from baseline as shown by the percent change. Alirocumab (MD –56.62%; 95% CI –60.70% to –52.54%; *p* < 0.001) and evolocumab (MD –68.10%; 95% CI –74.85% to –61.36%; *p* < 0.001) yielded the highest percent reduction in LDL-C from baseline when baseline LDL-C levels were between 70 mg/dL and 100 gm/dL, while the lowest percent reduction was observed for alirocumab (MD –37.26%; 95% CI –44.06% to –30.46%; *p* < 0.001) and evolocumab (MD –37.55%; 95% CI –40.47% to –34.63%; *p* < 0.001) in patients with baseline LDL-C levels of 160 mg/dL or greater. Alirocumab (*p* < 0.001, I-square = 94%) and evolocumab (*p* < 0.001, I-square = 93%) showed significant heterogeneities across the trials in the analyses of LDL-C; therefore, random-effect models were used. The results demonstrate that alirocumab and evolocumab exhibit distinct lipid-lowering effects at varying baseline LDL-C levels. This finding has significant implications for the development of personalized drug therapy.

### 3.5 Risk of Bias

**Fig. 7.**
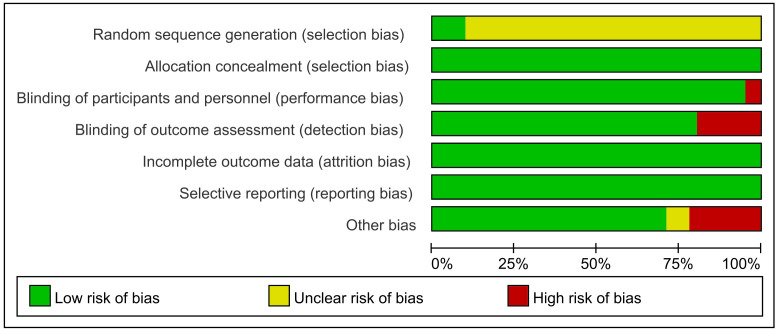
**Risk of bias graph**. The proportion of studies for each judgment (low risk, uncertain risk, high risk) for each entry in the tool was described.

**Fig. 8.**
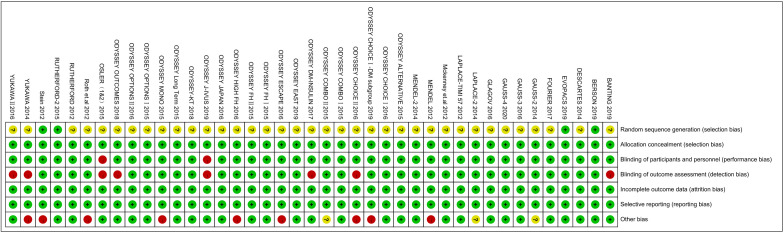
**Risk of bias summary**. A crosstab of judgment results for each entry of each study was described.

Fig. 7 includes a risk of bias graph, which shows the proportion of each judgement (low risk, high risk and uncertain risk) for each item in the tool for each study. Fig. 8 shows a risk of bias summary diagram, which represents a crosstab of judgement results for each item in each study [11]. In terms of individual studies, 4 or more items of each study were evaluated as having a low risk of bias. Most of the information stemmed from trials with a low risk of bias, and the included studies were not significantly different regarding risk of bias.

## 4. Discussion

Among these results, the incidence of cardiovascular death was lower in the group of alirocumab than in control. Nevertheless, alirocumab was statistically significant in reducing the risk of cardiovascular death only when baseline LDL-C was ≥100 mg/dL. The effect of evolocumab on cardiovascular mortality was not statistically significant for either baseline LDL-C levels below 100 mg/dL or above 100 mg/dL. According to our meta-analysis, both alirocumab and evolocumab presented a high efficacy in controlling lipids among various baseline LDL-C levels, and the percent changes in LDL-C from baseline during the follow-up period reflected substantial reductions of more than 50% with alirocumab and more than 60% with evolocumab. Furthermore, our analysis presents that alirocumab and evolocumab exhibit distinct lipid-lowering effects at varying baseline LDL-C levels.

Navarese *et al*.’s study [9] published in JAMA 2018 suggested that the optimal benefit from lipid-lowering therapy may be observed in patients with baseline LDL-C levels of 100 mg/dL or above. The differences were that our analysis mainly explored the benefit of alirocumab and evolocumab on cardiovascular mortality at different baseline levels, while Navarese *et al*. [9] elaborated on the association between less/more intensive LDL-C–lowering therapy and cardiovascular mortality. In the FOURIER trial [2], when detecting the outcomes of the cardiovascular endpoints individually, there was no significant difference in cardiovascular mortality or death from any cause between the 2 groups. Our findings also indicated that evolocumab did not have a significant effect on cardiovascular mortality. However, a reduction in cardiovascular events was found within the first year of evolocumab therapy. In the OSLER trial [45], differences in results may be due to the fact that OSLER trial was based on a relatively small number of events. In these studies [2, 39, 41, 45, 47, 48, 49], the follow-up of cardiovascular deaths of evolocumab ranged from 8 to 144 weeks, and the use of short follow-up periods in some trials resulted in insufficient demonstration of clinical benefits with evolocumab treatment. Furthermore, current management of cardiovascular events is more effective, which may account for the lack of mortality benefit. In our analyses, alirocumab significantly reduced the risk of cardiovascular mortality with a baseline LDL-C level of ≥100 mg/dL. This result is consistent with those of a series of ODYSSEY trials. The efficient management of blood lipids via alirocumab is the main reason for the reduced risk of cardiovascular mortality. In particular, alirocumab significantly reduced plasma levels of lipoprotein(a) (Lp(a)), which is an independent cardiovascular risk factor [53]. In the ODYSSEY FH I trial, the risk of cardiovascular events was reported to be 100-fold greater in patients with heterozygous familial hypercholesterolaemia (aged 20–39 years) than in the general population [24], which may be the reason why the benefit of cardiovascular mortality from alirocumab is not marked in familial hypercholesterolaemia. In the ODYSSEY LONG TERM trial, there was a 48% decrease in cardiovascular events observed in the alirocumab group; four patients in the alirocumab group died of coronary heart disease, and seven patients died in the control group. These discoveries preliminarily supported the hypothesis that alirocumab has the potential to offer cardiovascular outcome benefits in addition to its substantial LDL-C lowering capabilities [8]. For the lipid-lowering effect of alirocumab and evolocumab, our analyses generated results that were accordant with those of previous trials. In contrast, alirocumab and evolocumab yielded the highest percent reduction in LDL-C from baseline when baseline LDL-C levels were between 70 mg/dL and 100 gm/dL and the lowest reduction when baseline LDL-C levels were ≥160 mg/dL. The latest European Society of Cardiology guidelines recommended an LDL-C reduction of 50% or greater from baseline and an LDL-C goal of <70 mg/dL are recommended for patients at high CV risk [54]. Therefore, our findings can provide a preliminary reference for the clinical use of alirocumab and evolocumab.

The following limitations of our meta-analysis should be mentioned. First, most studies showed that alirocumab can significantly improve cardiovascular death events. However, alirocumab was not superior to the control in the outcome of cardiovascular mortality (RR 0.85; 95% CI 0.72 to 1.00; *p* = 0.06) in single drug analysis (not stratified by baseline), but the result of quantitative synthesis showed that it was more inclined to the intervention group. Second, the duration of follow-up is still relatively short for the treatment of cardiovascular adverse events, and longer-term trials are needed. Third, the open-label [35, 45] design of the trials could have influenced the reporting of cardiovascular death events. Fourth, the number of cardiovascular events in the partial RCTs was relatively small, which could limit test efficacy and increase the risk of type II errors. Hence, large-scale RCTs with long follow-up durations that elaborate on cardiovascular mortality and other adverse cardiovascular events are desperately needed.

## 5. Conclusions

According to the stratified exploration of baseline level of LDL-C and drug type, PCSK9 mAbs appeared different lipid-lowering efficacy and cardiovascular death benefit. Alirocumab was associated with a significant reduction in cardiovascular mortality at baseline LDL-C levels of ≥100 mg/dL. Evolocumab did not have a marked effect on cardiovascular mortality. Our findings appeared that alirocumab and evolocumab exhibit distinct lipid-lowering effects at varying baseline LDL-C levels. Alirocumab and Evolocumab presented a better lipid-lowering effect when the baseline level <100 mg/dL. The included trials exhibited no significant differences in regard to the risk of bias.

## Data Availability

All the data we extracted can be obtained from ClinicalTrials.gov and other published literature.

## Abbreviations

CV, cardiovascular; LDL-C, low-density lipoprotein cholesterol; PCSK9 mAbs, proprotein convertase subtilisin/kexin type 9 monoclonal antibodies; FH, familial hypercholesterolemia; HeFH, heterozygous FH; HoFH, homozygous FH; LDLR, LDL receptor; RCTs, randomized controlled trials; MI, myocardial infarction; HF, heart failure; CHD, coronary heart disease; DM, diabetes mellitus.

## Supplementary Material

Supplementary material associated with this article can be found, in the online version, at https://doi.org/10.31083/RCM26980.
