# Supplementary material for: Effects of Alirocumab and Evolocumab on Cardiovascular Mortality and LDL-C: Stratified According to the Baseline LDL-C Levels
Source: Rev Cardiovasc Med. 2025 Apr 25;26(4):26980. doi: 10.31083/RCM26980 (PMC12059789; doi:10.31083/RCM26980)
Supplement: Supplementary file 1 [file 2153-8174-26-4-26980-s1.zip › Supplementary Material-V1.docx]

**Supplementary Table1.Common statistical methods of meta-analysis**

| **Data type** | **Effect index** | **Fixed effects model** | **Random effects model** |
| --- | --- | --- | --- |
| **Categorical variables** | OR(odds ratio) | Mantel-Hanenszel(M-H) | DerSimonian-Laird |
|  |  | Inverse variance |  |
|  |  | Peto |  |
|  | RR(relative risk) | Mantel-Hanenszel(M-H) | DerSimonian-Laird |
|  |  | Inverse variance |  |
|  | RD(risk difference) | Mantel-Hanenszel(M-H) | DerSimonian-Laird |
|  |  | Inverse variance |  |
| **Numeric variables** | MD(mean difference) | Inverse variance | DerSimonian-Laird |
|  | SMD(standardized  mean difference ) | Inverse variance | DerSimonian-Laird |
