# Supplementary material for: Effects of Alirocumab and Evolocumab on Cardiovascular Mortality and LDL-C: Stratified According to the Baseline LDL-C Levels
Source: Rev Cardiovasc Med. 2025 Apr 25;26(4):26980. doi: 10.31083/RCM26980 (PMC12059789; doi:10.31083/RCM26980)
Supplement: Supplementary file 1 [file 2153-8174-26-4-26980-s1.zip › the PRISMA checklist-V3.docx]

| **Section and Topic** | **Item #** | **Checklist item** | **Location where item is reported** |
| --- | --- | --- | --- |
| **TITLE** | | |  |
| Title | 1 | Effects of Alirocumab and Evolocumab on Cardiovascular Mortality and LDL-C: Stratified according to the baseline level of LDL-C. | Title (pg1) |
| **ABSTRACT** | | |  |
| Abstract | 2 | Effects of Alirocumab and Evolocumab on Cardiovascular Mortality and LDL-C: Stratified according to the baseline level of LDL-C.  Background: A meta-analysis was conducted to assess whether cardiovascular mortality and lipid-lowering effect of alirocumab and evolocumab is affected by various baseline LDL-C levels.  Methods: PubMed, Ovid, Embase, and ClinicalTrials.gov were searched for literature published before June 2023. The trials were eligible for inclusion if they satisfied the following criteria:(1) population: adult patients with hypercholesterolemia or HeFH at high cardiovascular risk; (2) intervention: patients were treated with alirocumab or evolocumab; (3) control: patients who received other standard lipid-lowering drugs or placebo; (4) outcomes: percent changes in LDL-C from baseline, incidence of cardiovascular deaths; and (5) study design: phase II or III RCTs (Randomized Controlled Trials).The quality of each included trial was evaluated using the Cochrane Cooperative Network Bias Risk Assessment Tool, which includes seven criteria: random sequence generation (selection bias), allocation concealment (selection bias), blinding of participants and personnel (performance bias), blinding of outcome assessment (detection bias), incomplete outcome data (attrition bias), selective reporting (reporting bias), and other bias.Trials were categorized by drug type (alirocumab and evolocumab) and baseline LDL-C level. The primary endpoints were cardiovascular mortality and percent changes in LDL-C from baseline. Relative risks (RRs) and 95% CIs (confidence intervals) were used for categorical data (cardiovascular mortality). The mean difference (MD) and 95% CI of the percent change from baseline were used. The pooled effect estimates ascertain whether a fixed-effects or a random-effects model would be more appropriate by the test of heterogeneity. And then, the most suitable statistical method was then selected in accordance with the selected model.  Results: Forty-one RCTs were included in the meta-analysis. Evolocumab did not present a significant effect in the outcome of cardiovascular mortality whether the baseline data were greater than 100 mg/dl or less than 100 mg/dl. However, the Stratified result shows that alirocumab decreased the risk of cardiovascular mortality in patients with a baseline LDL-C level of ≥100 mg/dl (RR 0.45; 95%: CI: 0.22 to 0.92; P=0.03). In terms of lipid-lowering efficacy, alirocumab (MD -56.62%; 95% CI: -60.70% to -52.54%; P<0.001) and evolocumab (MD -68.10%; 95% CI: -74.85% to -61.36%; P<0.001) yielded the highest percent reduction in LDL-C when baseline LDL-C levels were 70-100 mg/dl, while the smallest reduction in alirocumab (MD -37.26%; 95% CI: -44.06% to -30.46%; P<0.001) and evolocumab (MD -37.55%; 95% CI: -40.47% to -34.63%; P<0.001) occurred with baseline LDL-C levels of ≥160 mg/dL.  Discussion (limitations and Implications): The following limitations of our meta-analysis should be mentioned. First, most studies showed that alirocumab can significantly improve cardiovascular death events. However, alirocumab was not superior to the control in the outcome of cardiovascular mortality (RR 0.85; 95% CI 0.72 to 1.00; P=0.06) in single drug analysis (not stratified by baseline), and the outcome of quantitative synthesis deviated. Second, the duration of follow-up is still relatively short for the treatment of cardiovascular adverse events, and longer-term trials are needed. Third, the open-label29, 39design of the trials could have influenced the reporting of cardiovascular death events. Fourth, the number of cardiovascular events in the partial RCTs was relatively small, which could limit test efficacy and increase the risk of type II errors. Our findings can provide a reference for the clinical use of alirocumab and evolocumab.The benefit of cardiovascular mortality may occur for patients with baseline LDL-C levels of 100mg/dl or greater (Alirocumab). However, the greatest benefit of lipid-lowering effect may occur for patients with baseline LDL-C levels of 70-100 mg/dL.  Funding: This work was supported by the National Natural Science Foundation of China (Project No.82000337); the Applied Basic Research Program of the Science and Technology Hall of Yunnan Province and Kunming Medical University (Project No. 202001AY070001-142); and the Applied Basic Research Program of the Science and Technology Hall of Yunnan Province and Kunming Medical University (Project No.202301AY070001-130). All authors were engaged in the research implementation and writing work of this study. Both funding and efforts are appreciated.  Register: We registered it with PROSPERO (CRD42023446723). | Abstract (pg1)  Methods (pg2-4)  Results (pg4, pg5, pg9)  Discussion (pg10-14)  Funding |
| **INTRODUCTION** | | |  |
| Rationale | 3 | Aggressive lipid management in high-risk cardiovascular patients can significantly improve cardiovascular outcomes. Statins represent the foundation of clinical lipid management. Nevertheless, for patients who are unable to achieve the targeted LDL-C levels with intensive statin therapy or for patients who are intolerant to statins, a combination of PCSK9 mAbs (proprotein convertase subtilisin/kexin type 9 monoclonal antibodies) may be employed as an alternative. A substantial body of evidence has demonstrated that statins are an effective intervention for reducing the incidence of cardiovascular events. Moreover, the combination of ezetimibe or PCSK9 mAbs with intensive statin therapy has been evidenced to result in a further reduction of LDL-C levels, thereby further declining cardiovascular risk. The current research mainly elaborated on the association between less/more intensive LDL-C–lowering therapy and cardiovascular mortality, and the benefits of alirocumab and evolocumab on cardiovascular mortality in patients with various baseline LDL-C levels are unclear. | Introduction (pg1-2) |
| Objectives | 4 | Effects of alirocumab and evolocumab on specific fatal and non fatal endpoints appear to vary in patients with various baseline LDL-C levels. Therefore, a meta-analysis was conducted to assess whether cardiovascular mortality and lipid-lowering effect of alirocumab and evolocumab is affected by various baseline LDL-C levels. | Introduction (pg2) |
| **METHODS** | | |  |
| Eligibility criteria | 5 | The trials were eligible for inclusion if they satisfied the following criteria:(1) population: adult patients with hypercholesterolemia or HeFH at high cardiovascular risk; (2) intervention: patients were treated with alirocumab or evolocumab; (3) control: patients who received other standard lipid-lowering drugs or placebo; (4) outcomes: percent changes in LDL-C from baseline, incidence of cardiovascular deaths; and (5) study design: phase II or III RCTs (Randomized Controlled Trials). | Methods (pg2) |
| Information sources | 6 | Two independent investigators HM and YL (Data curation)) conducted a comprehensive search of PubMed, Ovid, Embase, and ClinicalTrials.gov for articles published prior to June 2023. | Methods (pg2) |
| Search strategy | 7 | The key words of retrieval were “Proprotein convertase subtilisin/kexin type 9 inhibitor” OR “PCSK9 inhibitor” OR “PCSK9 monoclonal antibodies” OR “PCSK9 mAbs” OR “Alirocumab” OR “Evolocumab” OR “REGN727” OR “SAR236553” OR “AMG145” OR “RN316” OR “PF04950615” OR “IBI306”. In addition, we avoided possible omissions of eligible studies by searching the references of the review articles. | Methods (pg2) |
| Selection process | 8 | A total of 6713 records were retrieved from PubMed, Ovid, Embase, and ClinicalTrials.gov. A total of 215 records were accessed via full-text perusal after discarding duplicate records and removing irrelevant articles by scanning titles and abstracts. A total of 175 publications were excluded for the following reasons: the subject was not relevant (n=37); the intervention treatment did not include alirocumab or evolocumab (n=54); the participants included children (n=5); the publications were comments or case reports (n=6) or review articles (n=38); and adverse cardiovascular events (n=19) or outcomes (n=16) were not reported. Finally, 40 studies consisting of 41 RCTs were included in the meta-analysis. (The PRISMA flow diagram) | Figure1 |
| Data collection process | 9 | Data were independently extracted by two authors HM and YL (Data curation) and any divergences were resolved by the corresponding author. The information we extracted from the various studies was as follows: title of trials, date of publication, the registration number of clinical trial, baseline LDL-C mean, doses of alirocumab and evolocumab, the information of control group, background lipid-lowering treament, length of follow-up for blood lipids and adverse events, mean age, the proportion of patients with diabetes mellitus, the ratio of patients with coronary heart disease, and patient characteristics. | Methods (pg2) |
| Data items | 10a | For cardiovascular death events, we extracted the total amount of participants and the number of cardiovascular deaths from the studies. For the percent changes in LDL-C from baseline, we extracted the mean, standard deviation (SD), and the number of participants in each group. In the absence of reported standard deviations (SDs), these were calculated from the standard error or 95% confidence interval (CI). | Methods (pg2) |
|  | 10b | In the absence of reported standard deviations (SDs), these were calculated from the standard error or 95% confidence interval (CI). | Methods (pg2) |
| Study risk of bias assessment | 11 | The study quality of RCTs was assessed using the Cochrane Cooperative Network Bias Risk Assessment Tool, which includes seven criteria: random sequence generation (selection bias), allocation concealment (selection bias), blinding of participants and personnel (performance bias), blinding of outcome assessment (detection bias), incomplete outcome data (attrition bias), selective reporting (reporting bias), and other bias. | Methods (pg4) |
| Effect measures | 12 | The mean difference (MD) and 95% CI of the percent change from baseline were used. The pooled effect estimates ascertain whether a fixed-effects or a random-effects model would be more appropriate by the test of heterogeneity. And then, the most suitable statistical method was then selected in accordance with the selected model.The inspection level for pooled results was two-sided, and P<0.05 was considered to indicate statistical significance. Heterogeneity was evaluated using the chi-square heterogeneity statistic with P<0.05 considered to indicate statistical significance, and I-squared >50% was considered to indicate a high degree of heterogeneity. | Methods (pg4) |
| Synthesis methods | 13a | Eligible RCTs included adults with hypercholesterolemia or high cardiovascular risk who were treated with alirocumab or evolocumab and reported LDL-C changes and cardiovascular deaths. | Methods (pg2) |
|  | 13b | For cardiovascular death events, we extracted the total amount of participants and the number of cardiovascular deaths from the studies. For the percent changes in LDL-C from baseline, we extracted the mean, standard deviation (SD), and the number of participants in each group. In the absence of reported standard deviations (SDs), these were calculated from the standard error or 95% confidence interval (CI). | Methods (pg2) |
|  | 13c | Baseline data for each RCTS, especially baseline LDL-C and intervention in the trial, were summarized in tables for subsequent subgroup analysis. As for the results of subgroup analysis, the study was presented through the forest map of meta-analysis. | Table2 and Figures |
|  | 13d | Analyses were conducted using Review Manager 5.4 (Cochrane Collaboration). Relative risks (RRs) and 95% CIs (confidence intervals) were used for categorical data (cardiovascular mortality). The mean difference (MD) and 95% CI of the percent change from baseline were used. The pooled effect estimates ascertain whether a fixed-effects or a random-effects model would be more appropriate by the test of heterogeneity. And then, the most suitable statistical method was then selected in accordance with the selected model. The inspection level for pooled results was two-sided, and P<0.05 was considered to indicate statistical significance. Heterogeneity was evaluated using the chi-square heterogeneity statistic with P<0.05 considered to indicate statistical significance, and I-squared >50% was considered to indicate a high degree of heterogeneity. | Methods (pg3-4) |
|  | 13e | The study explored possible causes of heterogeneity by subgroup analysis. For research on cardiovascular mortality, we performed subgroup analysis according to drug type (alirocumab and evolocumab) and baseline LDL-C level(baseline LDL-C＜100 mg/dl and ≥100mg/dl ). For the percent changes in LDL-C from baseline, studies were grouped into four subgroups according to baseline LDL-C level (baseline LDL-C＜100 mg/dl, ≥100 and ＜130 mg/dl, ≥130 and ＜160 mg/dl, ≥160 mg/dl). | Methods (pg3-4) |
|  | 13f | Most of the RCTs included were large-scale, multicenter trials, and individual small sample studies had little impact on the results. | Table2 |
| Reporting bias assessment | 14 | The study quality of RCTs was assessed using the Cochrane Cooperative Network Bias Risk Assessment Tool which includes selective reporting (reporting bias). | Methods (pg4) |
| Certainty assessment | 15 | The heterogeneity test shows that the heterogeneity of the studies included in this paper is small. By formulating strict inclusion criteria, this paper ensures that documents with the same research purpose can enter and minimize the sources of heterogeneity. Heterogeneity was evaluated using the chi-square heterogeneity statistic with P<0.05 considered to indicate statistical significance, and I-squared >50% was considered to indicate a high degree of heterogeneity. | Methods (pg4) |
| **RESULTS** | | |  |
| Study selection | 16a | A total of 6713 records were retrieved from PubMed, Ovid, Embase, and ClinicalTrials.gov. A total of 215 records were accessed via full-text perusal after discarding duplicate records and removing irrelevant articles by scanning titles and abstracts. Finally, the meta-analysis was based on 40 studies, comprising 41RCTs. | Results (pg4) |
|  | 16b | A total of 215 records were accessed via full-text perusal after discarding duplicate records and removing irrelevant articles by scanning titles and abstracts. A total of 175 publications were excluded for the following reasons: the subject was not relevant (n=37); the intervention treatment did not include alirocumab or evolocumab (n=54); the participants included children (n=5); the publications were comments or case reports (n=6) or review articles (n=38); and adverse cardiovascular events (n=19) or outcomes (n=16) were not reported. | Results (pg4) |
| Study characteristics | 17 | Table 2 lists 41 RCTs included in the study. These RCTs were published between 2012 and 2020. The mean range of baseline LDL-C levels was 2.4 to 5.69 mmol/L (92.8 to 219.9 mg/dl), and further subgroup analysis was performed according to baseline levels. The follow-up period of blood lipids ranged from 8 to 192 weeks, while that for the evaluation of cardiovascular events spanned from 8 to 144 weeks across the included trials. The mean weighted age for participants across primary studies ranged from 49.6 to 64.4 years, and the proportions of patients with coronary heart disease (CHD) and diabetes mellitus (DM) were 3–100% and 0.16–100%, respectively. Most participants were diagnosed with hypercholesterolemia or heterozygous familial hypercholesterolemia, and the included patients of 2 RCTs were diagnosed with acute coronary syndrome. Background therapy was added with stable statin or other lipid-lowering therapy in most of the RCTs. | Results (pg4) Table2 |
| Risk of bias in studies | 18 | Figure7 includes a risk of bias graph, which shows the proportion of each judgement (low risk, high risk and uncertain risk) for each item in the tool for each study. Figure8 shows a risk of bias summary diagram, which represents a crosstab of judgement results for each item in each study.6 In terms of individual studies, 4 or more items of each study were evaluated as having a low risk of bias. Most of the information stemmed from trials with a low risk of bias, and the included trials were not significantly different regarding risk of bias. | Figure7,  Figure8 |
| Results of individual studies | 19 | Table 2 lists 41 RCTs included in the study.  Figure2. Cardiovascular mortality stratified by drug type (alirocumab and evolocumab). As shown in the two subgroups, alirocumab did not present a significant effect in the outcome of cardiovascular mortality (RR 0.85; 95% CI 0.72 to 1.00; P=0.06). However, the result of quantitative synthesis showed that it was more inclined to the intervention group. Cardiovascular deaths occurred in 1.93% (252/13,083) of participants in the alirocumab group and 2.51% (287/11,441) in the control group. Alirocumab exhibited a lower incidence of cardiovascular mortality.  Figure3. Cardiovascular mortality of alirocumab stratified by baseline LDL-C level. Cardiovascular mortality was markedly associated with a reduction in risk only in the trials with patients who had baseline LDL-C levels of 100 mg/dL or greater (RR 0.45; 95% CI 0.22 to 0.92; P=0.03, <0.05).  Figure4. Cardiovascular mortality of evolocumab stratified by baseline LDL-C level. We also stratified evolocumab based on baseline data, and there was still no difference in cardiovascular mortality whether the baseline data were greater than 100 mg/dl (RR 1.04; 95% CI 0.40 to 2.73; P=0.93) or less than 100 mg/dl (RR 1.04; 95% CI 0.87 to 1.24; P=0.65) (Figure4).  Figure5. Percent changes in LDL-C from baseline of alirocumab stratified by baseline LDL-C level.Figure6. Percent changes in LDL-C from baseline of evolocumab stratified by baseline LDL-C level. Alirocumab (MD -56.62%; 95% CI -60.70% to -52.54%; P<0.001) and evolocumab (MD -68.10%; 95% CI -74.85% to -61.36%; P<0.001) yielded the highest percent reduction in LDL-C from baseline when baseline LDL-C levels were between 70 mg/dl and 100 gm/dl, while the lowest percent reduction was observed for alirocumab (MD -37.26%; 95% CI -44.06% to -30.46%; P<0.001) and evolocumab (MD -37.55%; 95% CI -40.47% to -34.63%; P<0.001) in patients with baseline LDL-C levels of 160 mg/dL or greater. | Results (pg4, pg5, pg9)  Table2,  Figure2,  Figure3,  Figure4  Figure5,  Figure6, |
| Results of syntheses | 20a | In terms of individual studies, 4 or more items of each study were evaluated as having a low risk of bias. Most of the information stemmed from trials with a low risk of bias, and the included trials were not significantly different regarding risk of bias. | Results (pg9) |
|  | 20b | Alirocumab decreased the risk of cardiovascular mortality in patients with a baseline LDL-C level of ≥100 mg/dl (RR 0.45; 95%: CI: 0.22 to 0.92; P=0.03). However, evolocumab did not have a significant effect on cardiovascular mortality (RR 1.04; 95% CI: 0.88 to 1.24; P=0.65). Alirocumab (MD -56.62%; 95% CI: -60.70% to -52.54%; P<0.001) and evolocumab (MD -68.10%; 95% CI: -74.85% to -61.36%; P<0.001) yielded the highest percent reduction in LDL-C when baseline LDL-C levels were 70-100 mg/dl, while the smallest reduction in alirocumab (MD -37.26%; 95% CI: -44.06% to -30.46%; P<0.001) and evolocumab (MD -37.55%; 95% CI: -40.47% to -34.63%; P<0.001) occurred with baseline LDL-C levels of ≥160 mg/dL. | Results (pg4, pg5, pg9) |
|  | 20c | No marked heterogeneities were discovered in exploring cardiovascular mortality (I^2^=0%). Alirocumab (P<0.001, I^2^=94%) and evolocumab (P<0.001, I2=93%) showed significant heterogeneities between the trials in the analyses for LDL-C; therefore, random-effect models were used. | Results (pg4, pg5, pg9) |
|  | 20d | In the process of being included in the study, we explore the degree of influence on the results by eliminating the studies one by one. We find that after eliminating each study, there is almost no influence on the results, and the results of eliminating the studies one by one are more consistent. | NA |
| Reporting biases | 21 | Figure7 indicates that reporting biases were evaluated as having a low risk of bias. | Results (pg9)  Figure7 |
| Certainty of evidence | 22 | Quality evaluation of research can increase the credibility of evidence.In terms of individual studies, 4 or more items of each study were evaluated as having a low risk of bias. Most of the information stemmed from trials with a low risk of bias, and the included trials were not significantly different regarding risk of bias. | Results (pg9)  Figure7,Figure8 |
| **DISCUSSION** | | |  |
| Discussion | 23a | Navarese’s study published in JAMA 2018 suggested that the greatest benefit from LDL-C lowering therapy may occur for patients with baseline LDL-C levels of 100 mg/dL or greater. The differences were that our analysis mainly explored the benefit of alirocumab and evolocumab on cardiovascular mortality at different baseline levels, while Navarese elaborated on the association between less/more intensive LDL-C–lowering therapy and cardiovascular mortality. In the FOURIER trial, when detecting the outcomes of the cardiovascular endpoints individually, there was no significant difference in cardiovascular mortality or death from any cause between the 2 groups. Our findings also indicated that evolocumab did not have a significant effect on cardiovascular mortality. However, a reduction in cardiovascular events was found within the first year of evolocumab therapy In the OSLER trial. Differences in results may be due to the fact that OSLER trial was based on a relatively small number of events. In our analyses, alirocumab significantly reduced the risk of cardiovascular mortality with a baseline LDL-C level of ≥100 mg/dl. This result is consistent with those of a series of ODYSSEY trials. The efficient management of blood lipids via alirocumab is the main reason for the reduced risk of cardiovascular mortality. In particular, alirocumab significantly reduced plasma levels of Lp(a), which is an independent cardiovascular risk factor and is higher in patients with FH. In the ODYSSEY FH I trial, the risk of cardiovascular events was reported to be 100-fold greater in patients with heterozygous familial hypercholesterolaemia (aged 20–39 years) than in the general population,which may be the reason why the benefit of cardiovascular mortality from alirocumab is not marked in familial hypercholesterolaemia. In the ODYSSEY LONG TERM trial, there was a 48% decrease in cardiovascular events observed in the alirocumab group; four patients in the alirocumab group died of coronary heart disease, and seven patients died in the control group. These discoveries preliminarily supported the hypothesis that alirocumab has the potential to offer cardiovascular outcome benefits in addition to its substantial LDL-C lowering capabilities. | Discussion (pg10-14) |
|  | 23b | First, most studies showed that alirocumab can significantly improve cardiovascular death events. However, alirocumab was not superior to the control in the outcome of cardiovascular mortality (RR 0.85; 95% CI 0.72 to 1.00; P=0.06) in single drug analysis (not stratified by baseline), but the result of quantitative synthesis showed that it was more inclined to the intervention group. Second, the duration of follow-up is still relatively short for the treatment of cardiovascular adverse events, and longer-term trials are needed. Third, the open-label design of the trials could have influenced the reporting of cardiovascular death events. Fourth, the number of cardiovascular events in the partial RCTs was relatively small, which could limit test efficacy and increase the risk of type II errors. | Discussion (pg14) |
|  | 23c | In the search for related studies on PCSK9 inhibitors, we found that the exploration of cardiovascular death is relatively limited, the sample size of existing studies on cardiovascular mortality is also limited, and the specific causes of cardiovascular mortality are different, which may also involve the influence of other confounding factors. | NA |
|  | 23d | Our research investigated the lipid-lowering effect of alirocumab and evolocumab based on baseline lipid levels, and we also comprehensively analysed the effect of alirocumab and evolocumab on the important hard endpoint of cardiovascular death at different baseline LDL-C levels. This feature represents an innovation that sets it apart from previous research. Our fingings can provide a reference for the clinical use of alirocumab and evolocumab. Alirocumab significantly reduced the risk of cardiovascular mortality with a baseline LDL-C level of 100 mg/dl and greater. Evolocumab did not have a marked effect on cardiovascular mortality whether the baseline data were greater than 100 mg/dl or less than 100 mg/dl.The highest reduction percentage of LDL-C occurred in baseline LDL-C levels between 70-100 mg/dL, whereas the lowest percentage reduction was observed in patients with baseline LDL-C levels of 160 mg/dL and greater. | Discussion (pg10-14) |
| **OTHER INFORMATION** | | |  |
| (Registration and protocol | 24a | We registered it with PROSPERO (CRD42023446723). | Methods (pg2) |
|  | 24b | We registered the protocol in PROSPERO and got the CRD number. | Methods (pg2) |
|  | 24c | There was no amendment during the registration period. | NA |
| Support | 25 | This work was supported by the National Natural Science Foundation of China (Project No.82000337); the Applied Basic Research Program of the Science and Technology Hall of Yunnan Province and Kunming Medical University (Project No. 202001AY070001-142); and the Applied Basic Research Program of the Science and Technology Hall of Yunnan Province and Kunming Medical University (Project No.202301AY070001-130). | Funding |
| Competing interests | 26 | All authors have no conflict of interest to disclose. | Conflict of Interest |
| Availability of data, code and other materials | 27 | All the data we extracted can be obtained from ClinicalTrials.gov and other published literature. | Availability of Data and Materials |

*From:*  Page MJ, McKenzie JE, Bossuyt PM, Boutron I, Hoffmann TC, Mulrow CD, et al. The PRISMA 2020 statement: an updated guideline for reporting systematic reviews. BMJ 2021;372:n71. doi: 10.1136/bmj.n71

For more information, visit: <http://www.prisma-statement.org/>
